# Effects of *Pedicularis kansuensis* Expansion on Plant Community Characteristics and Soil Nutrients in an Alpine Grassland

**DOI:** 10.3390/plants11131673

**Published:** 2022-06-24

**Authors:** Ruimin Qin, Jingjing Wei, Li Ma, Zhonghua Zhang, Yandi She, Hongye Su, Tao Chang, Beilong Xie, Honglin Li, Wenying Wang, Guoxi Shi, Huakun Zhou

**Affiliations:** 1 Qinghai Provincial Key Laboratory of Restoration Ecology in Cold Regions, Northwest Institute of Plateau Biology, Chinese Academy of Sciences, Xining 810008, China; qinruimin@nwipb.cas.cn (R.Q.); 202147341023@stu.qhnu.edu.cn (J.W.); mali@nwipb.cas.cn (L.M.); zhangzhonghua@nwipb.cas.cn (Z.Z.); sheyandi@nwipb.cas.cn (Y.S.); suhongye@nwipb.cas.cn (H.S.); changtao@nwipb.cas.cn (T.C.); xiebl713@163.com (B.X.); honglinli@qhu.edu.cn (H.L.); 2 University of Chinese Academy of Sciences, Beijing 100049, China; 3 College of Geography Science, Qinghai Normal University, Xining 810008, China; 4 College of Eco-Environmental Engineering, Qinghai University, Xining 810016, China; 5 State Key Laboratory of Plateau Ecology and Agriculture, Qinghai University, Xining 810016, China; 6 College of Life Science, Qinghai Normal University, Xining 810008, China; wangwy0106@163.com; 7 College of Bioengineering and Biotechnology, Tianshui Normal University, Tianshui 741000, China; shigx1986@163.com

**Keywords:** *Pedicularis kansuensis*, population expansion, plant community, soil nutrient, alpine grassland

## Abstract

*Pedicularis kansuensis* is an indicator species of grassland degradation. Its population expansion dramatically impacts the production and service function of the grassland ecosystem, but the effects and mechanisms of the expansion are still unclear. In order to understand the ecological effects of *P. kansuensis*, three *P. kansuensis* patches of different densities were selected in an alpine grassland, and species diversity indexes, biomasses, soil physicochemical properties, and the mechanism among them were analyzed. The results showed that *P. kansuensis* expansion increased the richness index, the Shannon–Wiener index significantly, and the aboveground biomass ratio (ABR) of the Weed group (*p* < 0.05), but reduced the total biomass of the community and the ABR of the Gramineae and Cyperaceae decreased insignificantly (*p* > 0.05); soil moisture, soil AOC, and NO_3_^−^·N decreased significantly (*p* < 0.05), while soil pH and total soil nutrients did not change significantly, and available phosphorus (AP) decreased at first and then increased (*p* < 0.05). The structural equation model (SEM) showed that *P. kansuensis* expansion had a significant positive effect on the community richness index, and a significant negative effect followed on the soil AOC from the increase of the index; the increase of pH had a significant negative effect on the soil AOC, NO_3_^−^·N, and AP. It indicated that *P. kansuensis* expansion resulted in the increase of species richness, the ABR of the Weed group, and the community’s water demand, which promoted the over-utilization of soil available nutrients in turn, and finally caused the decline of soil quality. This study elucidated a possible mechanism of poisonous weeds expansion, and provided a scientific and theoretical basis for grassland management.

## 1. Introduction

Alpine grassland is one of the most widely distributed ecosystems on the Qinghai–Tibet Plateau (QTP), accounting for approximately 60% of the land area of the QTP [1,2], which is the material basis for the sustainable development of grassland animal husbandry [3]. Due to the special environment of its growth and its sensitivity to disturbance, alpine grassland has become an important factor indicating the security of the QTP ecosystem, and its production function also profoundly affects the economic development of regional pastoralism [4]. However, alpine grasslands have undergone different degrees of degradation under the direct and indirect impacts of climate change and human activities [2,5]. The degraded area reached 5.82 × 10^5^ km^2^ from 2001 to 2013, accounting for nearly 40% of the total alpine grassland area [5], and it significantly threatens the ecological security and economic development of local, national, and even Southeast Asia [6,7]. It is gratifying that grassland ecological protection and construction projects have promoted the grassland restoration, such as the policy of restoring grazing land to grassland and the policy of awards and subsidies, which resulted in the vegetation coverage and grass height in the project area increasing by 16.9% and 59.8%, respectively [6], benefiting soil animal diversity, microbial diversity, and the stability of the entire ecosystem indirectly [2]. Hence, the study of grassland degradation and grassland restoration needs to be strengthened.

Land degradation includes the decline of pasture productivity, fragmentation of the landscape, reduction of soil nutrients, soil compaction, an increase of poisonous weeds species, or a combination of several of these [8]. With the increase of degradation degree, total vegetation cover and total biomass show a gradual decline generally [9,10,11], and so do the species diversity indexes [10]. However, some studies concluded that total community cover, total biomass, and species diversity index reach the highest level at moderate degradation and then decline gradually with the shape of a unimodal curve [12]. Community species gradually shifted from perennial herbs (Gramineae and Cyperaceae) to annual herbs along a degradation gradient [13], with a decrease in elite forages and an increase of the invasive species abundance [14]. The index of the weed and the proportion of poisonous weeds in the community gradually increased [11,15] and finally formed a grassland community with a single dominance of poisonous weeds, which significantly impacted plant community species composition and ecosystem carbon and water cycles [14]. Soil physicochemical properties also tend to deteriorate in the degradation process, manifested by the decrease of soil moisture and compactness, the increase of bulk density [12], the decline of soil fertility, the increasing of barrenness, the growth of xerophytes or psammophytes in the heavy degradation stage, and the appearance of an initial sandy landscape [12,13]. As a particular form of grassland degradation, the study of the process and mechanism of the invasion and population expansion of toxic weeds is needed.

Endemic to Northwestern China, *P. kansuensis* is an annual or biennial herb of the genus *Pedicularis*, family Scrophulariaceae [16]. It became a poisonous weed in the process of alpine grassland degradation [17] and was recognized as the iconic plant in the reverse succession of natural grassland communities under the influence of climate change and human activities [17,18,19]. After the expansion of *P. kansuensis*, the dominant species were basically the same and did not change in the community composition, but the subdominant species and the main associated species diverged [20]. Meanwhile, the coverage, density, and biomass of the Gramineae forages were inhibited [18], and livestock dislike eating *P. kansuensis* with its poor palatability, both of which brought great harm to the local animal husbandry and affected the sustainable use of grassland [21,22].

In such a context, it is necessary to research the characteristics and mechanism of the vegetation–soil system in the process of *P. kansuensis* expansion. In this study, we took the alpine grassland of the QTP as the research object, selected different densities of *P. kansuensis* patches as different stages of its expansion process, and explored the change characteristics of plant communities, soil physicochemical properties, and influence mechanisms during the expansion. It is expected to reveal the key ecological processes of toxic weeds invasion in grassland degradation and provide theoretical support for preventing and controlling their invasion and expansion.

## 2. Materials and Methods

### 2.1. Study Site

**Figure 1:**
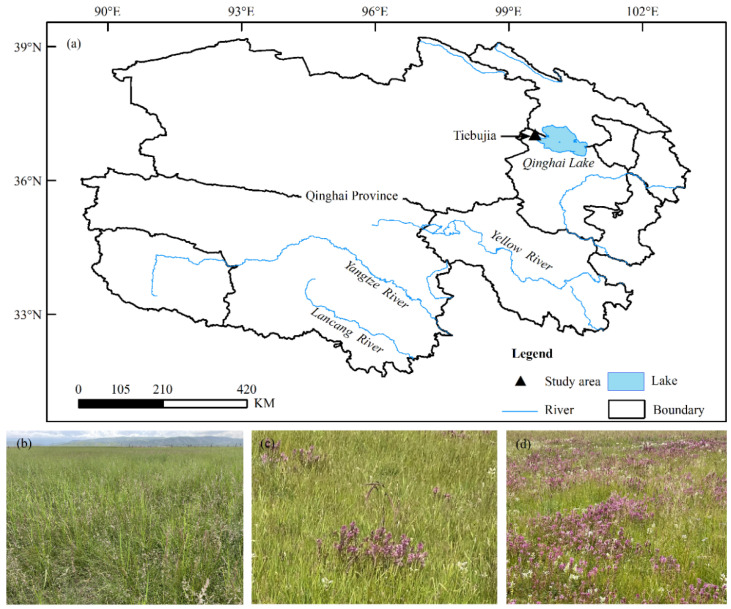
Location of the study site in Qinghai province (**a**), control plot patch (CK) (**b**), low density patch (LD) (**c**), and high density patch (HD) (**d**).

The experimental site (99°35′ N, 37°02′ E; 3270 m a.s.l.) is located at Tiebujia Grassland Improvement Experiment Station in Gonghe county, Hainan Tibetan autonomous prefecture, Qinghai province (Figure 1a). The climate is a typical plateau arid continental climate, with cool summers and cold winters, long sunshine time, and a large temperature difference between day and night. There is an average annual temperature of nearly 0 °C, annual precipitation of roughly 377 mm, and annual evaporation of about 1484 mm in the area [23]. The terrain of the alpine grassland is relatively flat, and plant communities are distributed evenly, with *Poa pratensis*, *Elymus breviaristatus*, and *Artemisia scoparia* as the main constructive species, and chestnut soil and dark chestnut soil as the primary soil [24].

### 2.2. Experimental Design

In early August 2020, three different density patches of *P. kansuensis*, no *P. kansuensis* (CK), low density (LD), and high density (HD) (Figure 1b–d), were selected to represent the three stages of population expansion in the flat grassland of the experimental area. The selection criteria of the patches are shown in Table 1. In each patch, five quadrats of 1 m × 1 m were selected using a completely random method, and the distance between each quadrat was at least 10 m. 

In each quadrat, the species characteristics survey included the species name, species number, total cover, abundance, height, frequency, and sub-cover of each species; aboveground biomass was harvested along the ground; and a soil drill with an inner diameter of 5 cm was used to drill the root–soil mixed samples, with each quadrat taking three drills along the diagonal, and each drill dividing into 0–10 cm, 10–20 cm, and 20–30 cm. Aboveground biomass (divided into the Gramineae, Cyperaceae, and Weed (other herbs except Gramineae and Cyperaceae) groups) was cut out, put into envelopes, and taken back to the laboratory to air dry. The root–soil mixed samples were put into plastic bags and taken back to the laboratory. Then, a 60-mesh standard soil sieve (pore size 0.28 mm) was used to separate the root and soil samples and they were rinsed and dried in the air; next, the aboveground biomass samples and root samples were put in a drying oven at 65 °C for 48 h to constant weight; and finally, electronic scales (JA2003N, Hangping, Shanghai, China) with an accuracy of 0.001 g were used to weigh the samples. 

Soil moisture was determined by measuring the fresh weight of the sample, then drying at 105 °C, and finally subtracting the dry weight from fresh weight [13]. Soil pH was measured using a pH meter (FE28K, Mettler Toledo, Shanghai, China) in the soil suspension of 1:5 soil/water (distilled water) [13]. Soil total carbon (TC) and total nitrogen (TN) were determined with an Elemental Analyzer (Elementar Vario, Langenselbold, Germany) [13]. Soil total phosphorus (TP) was colorimetrically assayed following the digestion of H_2_SO_4_ and HClO_4_ (UV-1800, Shimadzu, Japan), and available phosphorus (AP) was extracted by 0.5 mol/L NaHCO_3_ [13]. Soil total organic carbon (TOC) concentrations were evaluated using H_2_SO_4_–K_2_Cr_2_O_7_ oxidation and active organic carbon (AOC) using the KMnO_4_ oxidation method [13].

### 2.3. Statistical Analysis

Species diversity indicators selected in this study include the richness index, Shannon–Wiener diversity index, Simpson diversity index, and Pielou evenness index, and the calculation formulas are as follows:

(1) $$IV=\frac{ra+rh+rc+rf}{4}$$

(2) R=S

(3) $$H\prime =1-\overset{S}{\underset{i=1}{\sum }}p_{i}^{2}$$

(4) $$H=-\overset{S}{\underset{i=1}{\sum }}p_{i}\ln(p_{i})$$

(5) $$E=\frac{H}{\ln(S)}$$

(6) $$p_{i}=\frac{IV_{i}}{IV_{total}}$$

where *IV* is the important value, *ra* is the relative abundance, *rh* is the relative height, *rc* is the relative coverage, *rf* is the relative frequency, *R* is the species richness index, *H’* is the Simpson index, *H* is the Shannon–Wiener diversity index, *E* is the Pielou evenness index, *i* is the plant species of *i* in the sample box, and *S* is the total number of plant species in the sample box [25].

SPSS 26.0 software (SPSS Inc., Chicago, IL, USA) was used to analyze the significance of differences of plant community diversity indexes, biomasses, and soil properties, and the method was one-way ANOVA. Boxplots, histograms, and line graphs of the paper were drawn using OriginPro 2021 software (OriginLab Corp., Hampton, MA, USA). The construction and visualization of the SEM were carried out using the lavaan and the semPlot package of the R platform (R Development Core Team (Vienna, Austria), 2022; version 4.1.2).

## 3. Results

### 3.1. Responses of Plant Communities to P. kansuensis Expansion

#### 3.1.1. Change of Community Characteristics

**Figure 2:**
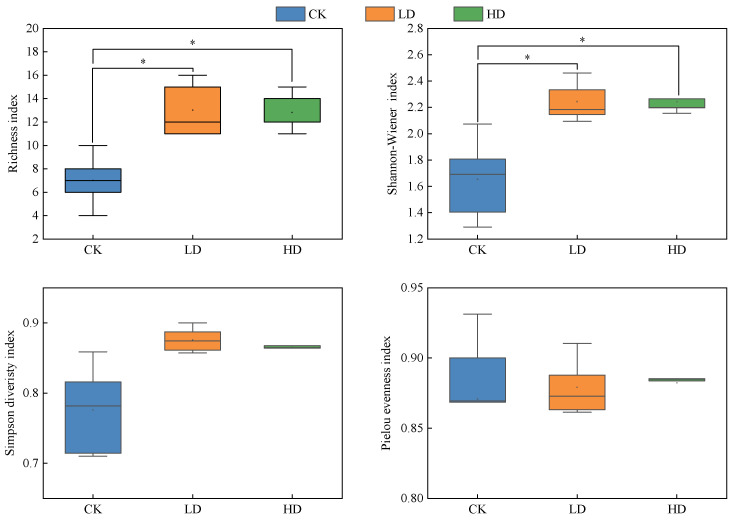
Communities’ diversity indexes of *P. kansuensis* patches in different densities; * indicates a significant difference at the 0.05 level.

With the density increase of *P. kansuensis*, the richness index and Shannon–Wiener index increased significantly, but the Simpson index and Pielou index did not change significantly. Compared with the CK patches, the richness index and Shannon–Wiener index of the LD and HD patches were significantly increased (*p* < 0.05), and the Simpson index and Pielou index just increased slightly (*p* > 0.05). Compared with the LD patches, the richness index, Shannon–Wiener index, and Simpson index of the HD patches decreased slightly, and the difference was not significant (*p* > 0.05), while the Pielou index increased slightly, and the difference was also not significant (*p* > 0.05) (Figure 2). 

#### 3.1.2. Change of Community Biomasses

**Figure 3:**
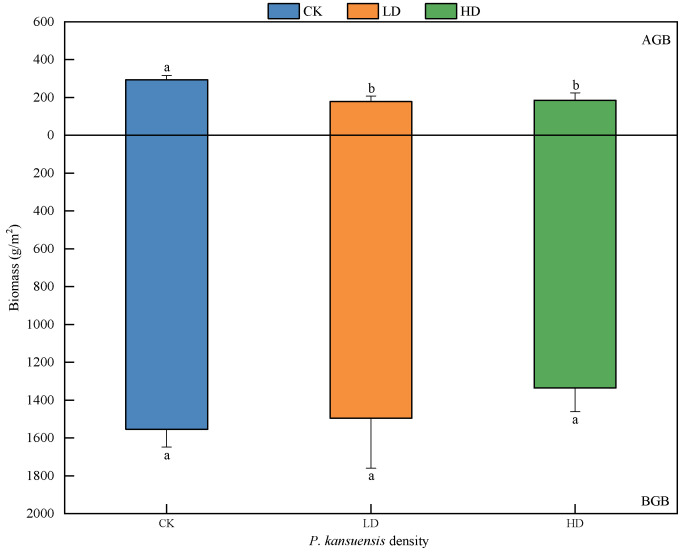
Community biomasses of *P. kansuensis* patches in different densities. AGB and BGB indicate aboveground biomass and belowground biomass, respectively. Various alphabetical characters depict significant differences among diverse density patches (*p* < 0.05).

With the density increase of *P. kansuensis*, the aboveground biomass of the community decreased significantly, while the belowground biomass decreased insignificantly. The aboveground biomasses of the CK patches, low-density patches, and high-density patches were 293.16 g/m^2^, 177.81 g/m^2^, and 184.55 g/m^2^, respectively, and the belowground biomasses were 1553.59 g/m^2^, 1494.95 g/m^2^, and 1336.25 g/m^2^, respectively. Compared with the CK patches, the aboveground biomass of the LD and HD patches decreased significantly (*p* < 0.05), and the belowground biomass only decreased slightly, and the difference was not significant (*p* > 0.05). Compared with the LD patches, the aboveground biomass of the HD patches increased slightly, and the difference was not significant (*p* > 0.05); the belowground biomass decreased slightly, and the difference was also not significant (*p* > 0.05) (Figure 3).

#### 3.1.3. Change of the ABR of Different Functional Groups

**Figure 4:**
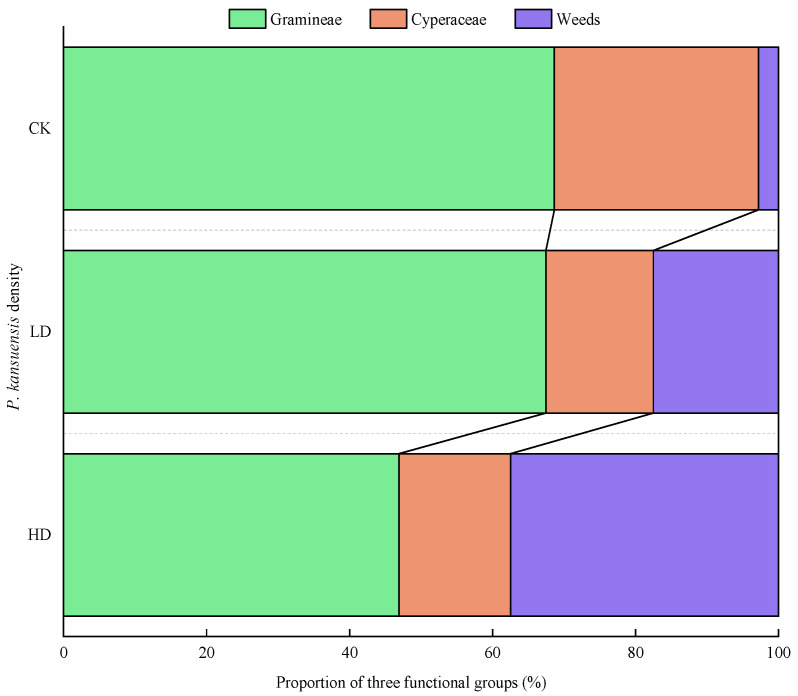
The ABR of different functional groups of *P. kansuensis* patches in different densities.

With the density increase of *P. kansuensis*, the ABR of the Gramineae and Cyperaceae decreased gradually, while the ABR of the Weed group increased significantly. Compared with the CK patches, the ABR of the Gramineae decreased by 1.18% and 21.71 in the LD patches and the HD patches, respectively, and the difference was not significant (*p* > 0.05). The ABR of the Cyperaceae decreased by 13.56% in the LD patches and 12.96% in the HD patches, and the difference was also not significant (*p* > 0.05). However, the ABR of the Weed group increased significantly by 14.73% (*p* < 0.05) and 34.70% (*p* < 0.05) in the LD patches and HD patches, respectively, indicating that the density increase of *P. kansuensis* negatively influenced the aboveground production of the Gramineae and Cyperaceae, but promoted the growth of the Weed group (Figure 4).

### 3.2. Responses of Soil Physicochemical Properties to P. kansuensis Expansion

#### 3.2.1. Change of Soil Moisture and pH

With the density increase of *P. kansuensis*, soil moisture decreased significantly as a whole, and the changing trend of soil moisture in the 0–20 cm soil layer was consistent. Compared with the CK patches, the soil moisture of the LD and HD patches decreased significantly by 5.87% (*p* < 0.05) and 10.74% (*p* < 0.05), respectively, in the 0–10 cm soil layer, and decreased significantly by 8.04% (*p* < 0.05) and 9.27% (*p* < 0.05), respectively, in 10–20 cm. In the 20–30 cm soil layer, soil moisture first decreased and then increased, and the soil moisture in the LD and HD patches was significantly lower than that in the CK patches, with a decrease of 10.82% (*p* < 0.05) and 8.35% (*p* < 0.05), respectively.

**Figure 5:**
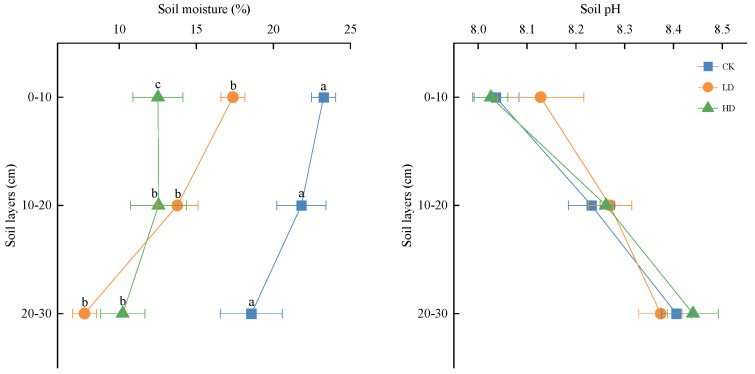
Soil moisture and pH of *P. kansuensis* patches in different densities. Various alphabetical characters depict significant differences among diverse density patches in the same soil layer (*p* < 0.05). The same applies to Figure 6.

The soil pH first increased and then decreased in the mass, and the difference was not significant (*p* > 0.05). Compared with the CK patches, the pH of the LD patches increased slightly in the 0–20 cm soil layer and decreased slightly in the 20–30 cm layer (*p* > 0.05); the pH of the HD patches decreased slightly in the 0–10 cm soil layer and increased slightly in the 10–30 cm layer (*p* > 0.05) (Figure 5).

#### 3.2.2. Change of Soil Nutrients

With the density increase of *P. kansuensis*, TC, TN, TOC, and NH_4_^+^·N showed a trend of first decreasing and then increasing. However, TP showed a decreasing trend, and the differences were all not significant (*p* > 0.05). Compared with the CK patches, the TC, TN, TOC, and NH_4_^+^·N in the LD patches decreased by 1.01 g·kg^−1^, 0.01 g·kg^−1^, 2.34 g·kg^−1^, and 0.06 mg·kg^−1^, respectively. The counterparts in the HD patches increased by 0.46 g·kg^−1^, 0.05 g·kg^−1^, 1.60 g·kg^−1^, and −0.01 mg·kg^−1^, respectively. Compared with the CK and LD patches, the TP decreased by 0.01 g·kg^−1^.

**Figure 6:**
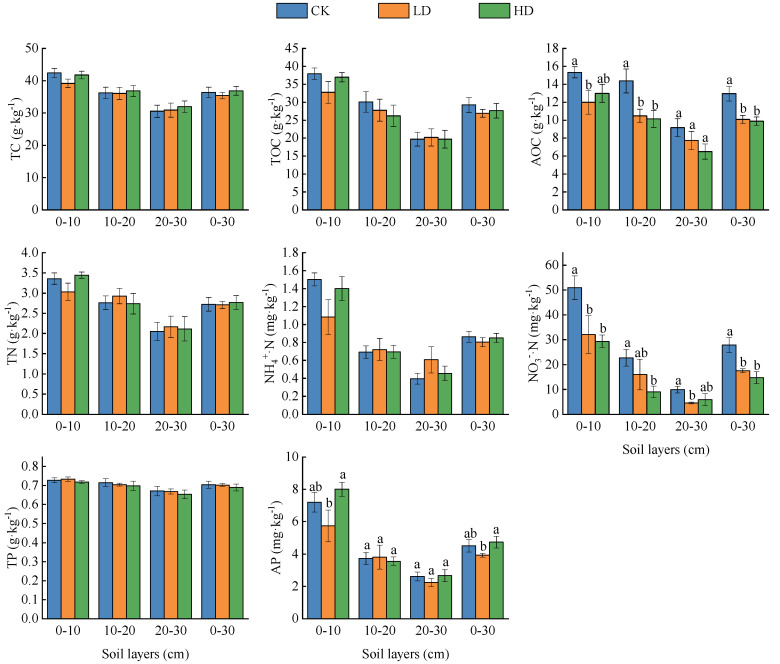
Soil nutrients of *P. kansuensis* patches in different densities.

Compared with the CK patches, the AOC gradually decreased in the LD and HD patches by 2.88 g·kg^−1^ (*p* < 0.05) and 3.08 g·kg^−1^ (*p* < 0.05), respectively. The variation of NO_3_^−^·N was consistent with that of AOC, decreasing by 10.34 mg·kg^−1^ (*p* < 0.05) and 13.12 mg·kg^−1^ (*p* < 0.05), respectively. AP decreased first and then increased with the density increase of *P. kansuensis*. The AP in the LD patches decreased by 0.49 mg·kg^−1^ (*p* > 0.05) compared with the CK patches, and the AP in the HD patches decreased by 0.81 mg·kg^−1^ compared with the LD patches (*p* < 0.05) (Figure 6).

### 3.3. Relationship between Community Characteristics and Soil Nutrients

**Figure 7:**
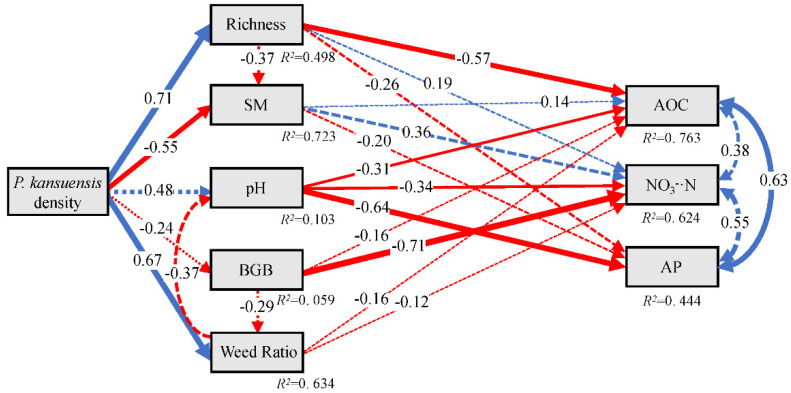
SEM of the mechanisms that related indicators affecting soil nutrients. The direction of the arrow indicates causality, the number on the arrow represents normalized path coefficient, and line thickness is positively correlated with significance; the solid blue line indicates a positive and significant relationship, the solid red line indicates a significant negative relationship, and the dashed line indicates a non-significant relationship. In the figure: treatment means three stages of *P. kansuensis* expansion; Richness means species richness; Weed Ratio means the weed ratio; BGB means belowground biomass; SM means soil moisture; pH means soil pH value; AOC means soil active organic carbon; NO_3_^−^·N means soil nitrate nitrogen; and AP means soil available phosphorus.

A structural equation model (SEM) was used to analyze how *P. kansuensis* expansion affected soil nutrients by affecting plant indicators and soil physical properties. The model fit the data in this study well (cfi = 0.929, srmr = 0.048, χ^2^/df = 1.516, *p* = 0.110). The results showed that the increase of the *P. kansuensis* density had a significant positive effect on the richness index and the Weed ratio, and a significant negative effect on soil moisture; then, the increase of the richness index developed a significant negative effect on soil AOC. Although there was no significant effect on belowground biomass and soil pH under the influence of *P. kansuensis* expansion, their changes developed a significant negative effect onNO_3_^−^·N, AOC, and AP (Figure 7).

## 4. Discussion

### 4.1. Community Characteristics and Biomass

In the process of alpine grassland degradation, the total community coverage is usually regarded as an essential indicator of the degradation stage [26], as is the emergence and expansion of some poisonous weeds [27,28]. In our study, however, we found that in the process of *P. kansuensis* expansion, the total community coverage did not change significantly, but the community composition and structure had changed. Among them, the richness index and Shannon–Weiner index increased significantly, indicating that the community was in a moderate degradation stage [12]. The growth and spread of the Weed group, *P. kansuensis* included, led to an increase of species number in the community, which reflected the rise and fall of different populations in the process of degradation and succession [12,26]. In terms of plant biomass, the AGB and BGB of the HD patches were reduced by 37.05% and 13.99% compared with the CK patches, respectively, which is consistent with previous research results [10,29,30], indicating that species richness and diversity indexes may temporarily increase after the invasion, but their biomass declines slowly. This may attribute to the reduction of the thickness and density of the grasses, the gradual vacancy of the ecological niches, and the decline of high-quality edible grasses such as the Gramineae [12,27,31]. The hemi-parasitic plant *P. kansuensis* obtains water and nutrients from the host plants by its haustoria to promote its growth [32], but it has a strong host preference [33]. In our study, the ABR of the Gramineae decreased by 15.61% in the HD patches and the ABR of the Cyperaceae by 15.45% in the LD patches, indicating that *P. kansuensis* may have been parasitized on the Gramineae during the early stages of the expansion, and absorbed a lot of water and nutrients in the subsequent expansion, leading to a rapid decline of the AGB of the Gramineae, which is similar to a study in Bayanbulak grassland [21], while the Cyperaceae may undergo this process during the early stages of the expansion. Furthermore, the parasitic preference of *P. kansuensis* to plant species needs more research.

### 4.2. Soil Physicochemical Properties

The degradation of alpine grassland will change the water and heat properties in shallow soil, affecting water and heat exchange on the surface and resulting in a potential change of the ecological environment [34]. In this study, with the density increase of *P. kansuensis*, soil moisture was significantly lower than that of the CK patches, which could be attributed to the increase in water demand after the increase in population density and community richness of *P. kansuensis*. In addition, it is also related to the enhanced evapotranspiration and infiltration caused by the reduction of the thickness and density of the grasses with the Weed invasion, which is consistent with the results of a meta-analysis [26]. The soil pH value has an overall upward trend with the density increase of *P. kansuensis*. However, the change is not significant, indicating that the pH is not significantly influenced by the process of population expansion [10,12,26]. In contrast, a study found that the pH increased significantly in a grassland invaded by *Stellera chamaejasme* [35], which may be related to the difference in interspecific association between different dominant species in various grasslands.

Soil C, N, and P are essential indicators of soil nutrients affecting plant growth, development, and physiological metabolism, which have a non-negligible regulating and driving effect on ecosystems [36]. At the same time, changes in the soil nutrients are also more obvious in response to grassland degradation [35]. Showing a trend of first decreasing and then increasing, soil TC and TOC did not change significantly in the process of *P. kansuensis* expansion, which may be caused by the stress of C utilization on the soil surface after the Weed invasion. Then, the C increased after population stabilization and eased C utilization [28]. Soil AOC decreased significantly in the process of *P. kansuensis* expansion, indicating that the changes in total nutrients did not change significantly in this process. However, its components may have already changed and then developed a corresponding impact on the grassland ecosystem. Soil nitrogen comes from the organic matter decomposed and synthesized by plant residues in soil and determines the carbon content to a certain extent [37], so it is consistent in the change of soil carbon and nitrogen; it was proved in our study that the trends of soil TN and NH_4_^+^·N are the same as those of TC and TOC. The soil NO_3_^−^·N decreased significantly during the population expansion of *P. kansuensis*, indicating that the expansion enhanced its productivity and absorbed more abundant nitrogen [38], which caused more easily absorbed NO_3_^−^·N to become the primary source of nitrogen nutrients [39]. It is basically consistent with characteristics of available nitrogen changing with the degradation gradient [28] and the results of a meta-analysis [26]. Soil TP did not change significantly during the expansion. In contrast, the AP changed significantly, decreasing at first and then increasing, indicating that *P. kansuensis* requires many nutrients in the initial stage of the invasion. The nutrient utilization moderated after the population stabilized. It is coupled with the increase of litter and decomposed matter to increase its content, consistent with a study of *Ligularia virgaurea* [28]. As an annual or bi-annual weed, *P. kansuensis* may significantly enhance nutrient availability and cycling in the litter layer [40], which could confirm the increase of TC and TOC of the topsoil in the HD patches. Notably, its seeds were stored in the soil due to its strong reproductive capacity [17], and germinated after constructing artificial grasslands in degraded grasslands, which became one of the reasons why artificial grassland was invaded by *P. kansuensis*.

### 4.3. Mechanism Analysis of the P. kansuensis Expansion

We constructed the SEM model of *P. kansuensis* expansion based on the results above. During the process of *P. kansuensis* expansion, edible herbs ratio such as the Gramineae decreased and vacated the ecological niches [12,27], which led the Weed group to settle into the community, resulting in a significant increase in species richness to promote the growth of plants and the absorption of AOC; the biomass of the Weed group also increased significantly in this process. On the contrary, soil moisture decreased significantly despite community coverage almost not changing, indicating that *P. kansuensis* absorbed much water in the soil with its strong seed reproduction ability and cluster distribution [17]. Although there was no significant change in soil pH, it developed a significant negative effect on soil AOC, NO_3_^−^·N, and AP, indicating that the pH change was closely related to available nutrients in the soil. Its slight change may profoundly affect the soil chemical processes [41,42], which should be further studied. Meanwhile, BGB also had a significant and negative effect on soilNO_3_^−^·N, reflecting a negative correlation between the increase of root biomass and its absorption of soil nutrients. Overall, after the expansion process of *P. kansuensis*, the plant community, soil moisture, and other factors were affected significantly. Although no significant effect happened in the total soil nutrients, the components may have actually changed, which should be the focus and issue in our future research.

## 5. Conclusions

*P. kansuensis* expansion increased the species diversity, richness of the community, and ABR of the Weed group, and the plant community transferred from a community with dominant species of the Gramineae and Cyperaceae to a weed community dominated by *P. kansuensis*; soil moisture, AOC, NO_3_^−^·N, and AP decreased significantly, while soil pH and total nutrients did not change significantly, which indicated that *P. kansuensis* expansion could indirectly promote the absorption and utilization of soil available nutrients, and eventually lead to the decline of soil quality and grassland degradation. In grassland management, some ecological indexes confirmed in this study can be used to predict the expansion of poisonous weeds, such as community richness, weed ratio, and soil moisture. One or several physical, chemical, and biological measures such as manual removal, ploughing, and spraying chemicals should be used as soon as poisonous weeds occur to avoid their negative effects.

## Figures and Tables

**Table 1:** Selection of *P. kansuensis* under different densities.

Patch	Number of *P. kansuensis*	Coverage of *P. kansuensis* (%)	Community Coverage (%)
CK	0	0	93.8 ± 0.9
LD	3.8 ± 0.8	6.2 ± 1.8	92.6 ± 1.2
HD	14.4 ± 1.7	20.2 ± 1.3	91 ± 2.4

Values represent mean ± SD (*n* = 5).

## Data Availability

Not applicable.
